# Study protocol: parents as pain management in Swedish neonatal care – SWEpap, a multi-center randomized controlled trial

**DOI:** 10.1186/s12887-020-02356-7

**Published:** 2020-10-12

**Authors:** Emma Olsson, Martina Carlsen Misic, Randi Dovland Andersen, Jenny Ericson, Mats Eriksson, Ylva Thernström Blomqvist, Alexandra Ullsten

**Affiliations:** 1 Faculty of Medicine and Health, School of Health Sciences, Örebro University, S-701 82 Örebro, Sweden; 2 Department of Pediatrics, Faculty of Medicine and Health, Örebro University, Örebro, Sweden; 3 Department of Research, Telemark Hospital Trust, Skien, Norway; 4 Research Center for Habilitation and Rehabilitation Services and Models (CHARM), Institute of Health and Society, University of Oslo, Oslo, Norway; 5 School of Education, Health and Social Studies, Dalarna University, Falun, Sweden; 6 Center for Clinical Research Dalarna, Uppsala University, Falun, Sweden; 7 Department of Pediatrics, Falun Hospital, Falun, Sweden; 8 Neonatal Intensive Care Unit, University Hospital, Uppsala, Sweden; 9 Department of Women’s and Children’s Health, Uppsala University, Uppsala, Sweden; 10 Centre for Clinical Research, Region Värmland, Karlstad, Sweden

**Keywords:** Infants, Parents, Pain management, Skin-to-skin contact, Breastfeeding, Lullaby singing, Newborn metabolic screening, Randomized controlled trial

## Abstract

**Background:**

During the first period of life, critically ill as well as healthy newborn infants experience recurrent painful procedures. Parents are a valuable but often overlooked resource in procedural pain management in newborns. Interventions to improve parents’ knowledge and involvement in infants’ pain management are essential to implement in the care of the newborn infant. Neonatal pain research has studied a range of non-pharmacological pain alleviating strategies during painful procedures, yet, regarding *combined* multisensorial parent-driven non-pharmacological pain management, research is still lacking.

**Methods/design:**

A multi-center randomized controlled trial (RCT) with three parallel groups with the allocation ratio 1:1:1 is planned. The RCT “Parents as pain management in Swedish neonatal care – SWEpap”, will investigate the efficacy of combined pain management with skin-to-skin contact, breastfeeding and live parental lullaby singing compared with standard pain care initiated by health care professionals, during routine metabolic screening of newborn infants (PKU-test).

**Discussion:**

Parental involvement in neonatal pain management enables a range of comforting parental interventions such as skin-to-skin contact, breastfeeding, rocking and soothing vocalizations. To date, few studies have been published examining the efficacy of combined multisensorial parent-driven interventions. So far, research shows that the use of combined parent-driven pain management such as skin-to-skin contact and breastfeeding, is more effective in reducing behavioral responses to pain in infants, than using the pain-relieving interventions alone. Combined parental soothing behaviors that provide rhythmic (holding/rocking/vocalizing) or orogustatory/orotactile (feeding/pacifying) stimulation that keep the parent close to the infant, are more effective in a painful context. In the SWEpap study we also include parental live lullaby singing, which is an unexplored but promising biopsychosocial, multimodal and multisensory pain alleviating adjuvant, especially in combination with skin-to-skin contact and breastfeeding.

**Trial registration:**

ClinicalTrials.gov (NCT04341194) 10 April 2020.

## Background

Parents are a valuable but often overlooked resource in procedural pain management in newborn infants. The influence of the parent is particularly salient in early infancy and in extremely distressful situations such as painful procedures, because infants lack the resources to successfully self-regulate. Therefore, infants need an emotionally available and stable parent who responds adequately to the infant’s distress signals and who is able to soothe, regulate and share the infant’s states [1]. In the Nordic countries today, the concept of family-centered care (FCC) is considered best practice in the care of the newborn [2, 3]. Since the 1990s, FCC has been and still is, part of an ongoing paradigm shift where family involvement in the infant’s care and the parent-infant relationship are of central importance, a cornerstone in current neonatal and pediatric health care [4, 5]. Nonseparation of parents and infants is a protective measure in decreasing stress in both parents and infants [6] and should also be applied in painful procedures [7].

As part of routine postnatal care, all infants experience recurrent painful procedures early in life with blood sampling for newborn metabolic screening and immunization programs. Infants in the neonatal intensive care unit (NICU) receive the highest amount of pain exposure, on average between 7 and 17 painful procedures per day [8]. Far from all infants receive adequate pain management during procedures (Shah & Siu 2019, [9, 10]). Untreated pain leads to unnecessary suffering but also leaves the newborn infant at risk for long term negative consequences from the pain. Research have shown detrimental effects of repeated painful experiences in the newborn infant including altered cortical development [11, 12], altered pain processing, increased internalizing behavior [13] and long-term effects on cortisol response [14]. For infants born preterm, neonatal pain-related stress was associated with alterations in both early and in later developmental outcomes [15].

During the last decade, parents’ participation in infant pain management has become a focus for research in nursing pain science (e.g. [16–20]). Parental presence has been shown to increase documentation of nursing pain assessment and of the use of non-pharmacological pain-relieving methods such as skin-to-skin contact (SSC) and breastfeeding, and decrease the infant’s pain intensity and behavioral distress [21, 22]. Parents have a unique knowledge and perspective of their infant’s needs and personality and can be effective partners in the care of the infant, including pain management, if they are acknowledged by the health professionals [20, 23]. Not all parents are able to provide emotional and physical closeness, many parents do not know how to effectively comfort their child during a medical procedure, and some do not feel confident doing so [24]. But most parents experience a feeling of helplessness in their inability to protect their infant from pain during procedures [25, 26]. Therefore, health professionals need to safeguard parent-infant proximity, reciprocity, and establishment of parental responsibility, which are all essential factors for the parent-infant attachment process [26]. Interventions to improve parents’ knowledge and involvement in infants’ pain management are essential to implement in the care of the newborn infant [26]. Parents need to and want to participate actively in their infant’s pain management, and parents should receive education and guidance in various format, not just verbal information, on how to mitigate their infant’s pain [23, 24, 27–31].

Pain alleviating pharmacological agents such as oral sweet solutions, i.e. sucrose and glucose, are today part of standard care in neonatal and postnatal care and are considered to have a calming and analgesic effect on infants during painful procedures [32]. Sweet solutions have been extensively investigated and found to reduce procedural pain from blood-sampling procedures in preterm, term infants and infants ≤12 months old, without serious side effects [33]. However, there is a consensus that non-pharmacological strategies should be the first choice in procedural pain management for infants because there are no adverse effects and parents can be involved using these approaches safely [23, 34, 35]. Non-pharmacological pain management involves interventions driven by nurses and/or parents. Just like oral sweet solutions, swaddling, containment or facilitated tucking, positioning, non-nutritive sucking and recorded music stimulation, are nurse-initiated pain alleviating interventions where parents can be included but parental presence is not required [32, 36]. Among the non-pharmacological approaches, the biopsychosocial perspective strongly supports parent-driven interventions [37]. In the parent-driven non-pharmacological interventions, the parent herself/himself is a mediator for pain relief [36]. Parent-driven non-pharmacological interventions are consistent with modern family-centered care in which the best interests of the infant and family are put ahead of staff convenience [36]. SSC, breastfeeding and live singing are simple and cost-effective evidence-based interventions that may modify the infant’s pain and stress if the strategies are well-timed [38–41]. SSC is a method widely used in postnatal care globally. A significant amount of research has also shown the pain-relieving effect of skin-to-skin contact during painful procedures in newborn infants [19, 42]. Breastfeeding has demonstrated efficacy that is equal to, or greater than, sweet taste interventions in reducing behavioral and physiological responses to pain in full-term infants undergoing venipuncture with no demonstrated adverse outcomes [38]. Live parental infant-directed lullaby singing, informed by music therapy research, has not been previously investigated during painful procedures. It has been confirmed in previous research in non-painful contexts that live parental lullaby singing is an individually tailored, non-verbal, multisensory, affective, relationship-based tool useful in regulating the infant, augmenting the parent’s focus on the infant in the moment, enhancing the parent’s emotional availability and responsiveness and decreasing stress in the dyad [43–45]. Previous research with recorded music stimulation with instrumental lullaby music, selected in consultation with an accredited music therapist, has shown similar effect to the current gold standard of oral sucrose [46], and the combination of music stimulation with sucrose provides better pain relief during blood sampling than when sucrose or music is used alone [46]. Recorded mother’s voice has also shown similar pain alleviating effects as oral sweet solutions [47, 48].

Neonatal pain research has studied a range of non-pharmacological pain alleviating strategies during painful procedures. However, there is a dearth of research about combined multisensorial parent-driven non-pharmacological pain management, which encompasses a combination of individual pain-relieving interventions such as parental closeness and SSC, olfactory, oral and auditory stimulation. Therefore, a multi-center randomized controlled trial with the aim to investigate the efficacy of combined pain management with SSC, breastfeeding and live parental lullaby singing, is planned in postnatal care.

## Methods: hypothesis, participants, interventions, measures and outcomes

### Hypothesis

No previous research has assessed the efficacy of combined parent-driven pain management with SSC, breastfeeding and live parental lullaby singing in newborn infants during routine newborn metabolic screening. In this research study, “**P**arents **a**s **p**ain management in **Swe**dish neonatal care – **SWEpap**”, we hypothesize that parent-driven pain management such as SSC in combination with breastfeeding and live parental lullaby singing, will provide a more effective pain management during venipuncture in newborn infants compared to standard pain care initiated by health care professionals.

### Study design

This is a multi-center, randomized controlled trial (RCT) with three parallel groups with the allocation ratio 1:1:1.

### Study setting

This RCT involves parent-infant dyads recruited from four health care regions in Sweden during the routine newborn metabolic screening. In the Swedish context, the healthy newborn infant is often discharged soon after birth and returns at 48 h of age for a newborn screening test and medical examination. The involved regions represent both university hospitals and regional hospitals.

### Eligibility criteria

Healthy newborn infants, who will be screened with the routine newborn metabolic screening test and their parent are enrolled. Infants treated with sedatives or analgesics within the last 24 h are excluded. A written informed consent is acquired from the infant’s parents. Parents who understand Swedish or English are eligible for inclusion. The infant-parent dyad is randomly assigned to one of three treatment groups; one standard of care group with glucose (*n* = 75), one group with skin-to-skin contact (n = 75), and one group where skin-to-skin contact is combined with breastfeeding (if applicable) and live parental lullaby singing (n = 75), (Table 1).

**Table 1:** Treatment groups

Group	Treatment	Treatment description
Group 1	Standard care with glucose	The infant is placed on the examination table for the blood test. Standard care comprises facilitated tucking done by a nurse or the parent, 1–2 ml of oral glucose (300 mg/ml) given in fractioned doses from 2 min before the procedure and the opportunity to suck on a pacifier or on a parent’s or a nurse’s plastic gloved finger.
Group 2a	Parent-driven pain management with skin-to-skin contact	The parent will sit in an adjustable recliner chair during the procedure and the infant will be placed naked (except for a diaper and possibly a hat) on the parent’s bare chest 10 min before the venipuncture. The skin-to-skin contact will proceed during and a while after the procedure.
Group 2b	Parent-driven pain management with skin-to-skin contact and breastfeeding	The parent will sit in an adjustable recliner chair during the procedure and the infant will be placed naked (except for a diaper and possibly a hat) on the parent’s bare chest 10 min before the venipuncture. Breastfeeding starts about 2 min before the venipuncture and the blood test is performed when the infant is latched and sucking well.
Group 3	Parent-driven pain management with a combination of skin-to-skin contact, breastfeeding and live parental lullaby singing	The intervention in the combination group will follow the above described skin-to-skin and breastfeeding treatment descriptions. The parents are also instructed to hum a lullaby with their infant. The parent starts humming the lullaby when the infant is placed on the parent’s bare chest 10 min before the venipuncture and continues during and a while after the procedure. The humming should be performed in a simple, repetitive, soft and sedative mode in a low pitch, in consonant harmony, with a slow, steady and predictable pulse of 3/4 or 6/8 rhythm, maintaining a constant sound level between recommended ≤55–65 dB on the A-scale [49].

### Interventions

The treatment groups in the RCT are described in Table 1. Group 2, comprising SSC, is divided into two subgroups, one with only SSC and one where the mother also chooses to breastfeed her infant during the test. The reason for these subgroups is that standard of care in one of the hospitals does not include glucose but comprises only SSC and breastfeeding when applicable. To ensure intervention fidelity in the live parental lullaby singing, a short video, showing a parent who sings according to the treatment description of the lullaby singing, will be played for the parents who are randomly assigned to group 3. These parents will also be verbally guided in how to sing before the intervention starts. The verbal instructions are simplified and delivered in dialogue with the parent who can ask questions.

### Outcomes

The primary outcome is infant pain expression measured with Premature Infant Pain Profile Revised (PIPP-R) [50], which is a pain assessment instrument that has also been translated into Swedish [51].

Secondary outcomes in the RCT are: a) changes in galvanic skin response (GSR) (area small peaks, area huge peaks, peaks per second, average rise time, average peak), which is obtained via three electrodes on the infant’s foot [52], b) parents’ rating of the infant’s pain, c) parents’ rating of their own stress during the procedure and d) parents’ rating of how beneficial the pain management they were involved in felt to them and their infant.

### Study measures

The PIPP-R has been tested for reliability, construct validity and clinical utility, all with results indicating good psychometrics [50]. The PIPP-R evaluates three behavioral facial actions (brow bulge, eye squeeze and nasolabial furrow), two physiological items (heart rate, oxygen saturation), and two contextual items (gestational age and behavioral state). The PIPP-R gives a weighted and higher score for the youngest infants to adjust for their lesser ability to show signs of pain. Scores can range from 0 to 21 where a higher score signifies a higher level of rated pain. A PIPP-R pain assessment includes a 15 s baseline measurement of heart rate and oxygen saturation together with an observation of the infant’s behavioral state and gestational age. Changes in physiological and behavioral indicators from baseline are then assessed during the first 30 s of the painful procedure. PIPP-R scores will be assessed from video recordings after the procedure by a trained nurse and a subset will also be assessed by one of the researchers for interrater reliability [50].

Galvanic skin response (GSR) refers to changes in sweat gland activity in response to a sensory stimulus. GSR measurements detects changes in electrical (ionic) activity resulting from changes in sweat gland activity. The increase in skin conductance reflects the infant’s arousal intensity in response to the sensory stimulus. As an indicator of pain, skin conductance measurements have detected increased sweating in newborn infants < 28 + 0 postnatal age submitted to heel lancing [53]. GSR have been tested in several neonatal pain studies on term and preterm infants, measuring skin conductance that reflects pain-related activation of the sympathetic nervous system [52–55]. Analyses of GSR are presented with the following variables; area small peaks, area huge peaks (both representing forcefulness of sympathetic nerve firing), peaks per second (the rate of firing in the sympathetic nerves), average rise time, average peak. Higher values in any of the above GSR parameters are indicative of more stress [52].

For the parents’ ratings, a visual analogue scale (VAS) with a 10-cm line anchored at the ends is used; from “no pain” on the left end point, up to “worst imaginable pain” on the right end point, from “no stress” on the left end point, up to “worst imaginable stress” on the right end point and finally from “not beneficial” on the left end point, up to “most beneficial possible” on the right end point of the VAS scale. The parents will perform the rating on the VAS scales right after the blood sampling procedure.

### Sample size

No previous RCT has examined the effects of parent-driven pain management in newborn infants by combining SSC, breastfeeding and live parental lullaby singing. Based on previous studies using PIPP-R or its predecessor PIPP in pain alleviation projects, a difference of two PIPP-R points between standard care (group 1) and the full parent driven pain management (group 3) is considered clinically important. Based on those studies we also assume a standard deviation around 2 points. A power calculation sets the number of infants to include in the study to 63 in each group, with a power of 0.8 and a significance level set to 0.05 (www.clincalc.com). To compensate for possible dropouts, incomplete data, such as equipment malfunction or blood sample collection failure, 75 infants per group will be enrolled, making a total of 225 infants.

### Recruitment and allocation

**Fig. 1:**
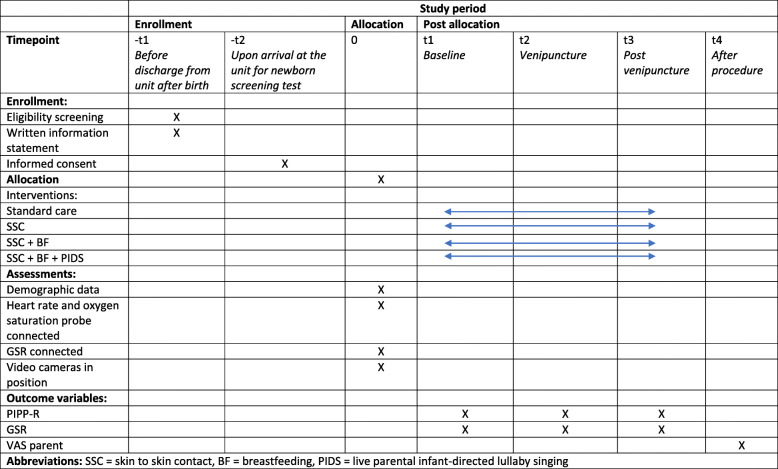
Timeline for the SWEpap study

The researcher in each region will present the study and provide written information about the study to eligible parents before they are discharged from the hospital (Fig. 1. Timeline SWEpap study). When the parent-infant dyads return to the postnatal care unit for the metabolic screening test, they will be asked to participate and to sign the consent form (both the mother and her partner). The parents who have accepted to participate are then randomly assigned to one of the three interventions groups using the sealed opaque envelope system with randomly generated treatment allocations administered by an impartial researcher. Once the parents have consented to enter the trial, the researcher opens the envelope and the allocated treatment regimen is offered to the parents. The researchers will then guide the parents in each group how to position themselves and the infant and if applicable how to use live parental lullaby singing. If the parent does not adhere to the protocol in the allocated treatment group, the infant will be excluded from the study.

Blinding will not be possible for the nurse and parents during the procedures. Since only the infant’s face and pulse oximetry will be videotaped, the subsequent PIPP-R pain assessment and GSR measurements will be blinded using muted recordings. The parents’ participation in the study is voluntary and they are informed in advance that they can withdraw at any time without giving any reason.

## Methods: data collection, data analyses and management, data monitoring

### Data collection

Demographic data of the parent-infant dyad is registered before the procedure starts, including registration of potential music and singing activities during pregnancy for the parents in group 3. In all groups: before, during and after the procedure the infant will have a saturation probe attached to one foot making it possible to measure heart rate and oxygen saturation and electrodes measuring galvanic skin response on the other foot. The infant’s face and pulse oximetry values will be videotaped for later pain assessment with PIPP-R. An experienced midwife/nurse will perform the venipuncture. Venipuncture is routinely used in all hospitals in Swedish neonatal and postnatal care since it is considered less painful for the infant than heel lance [56]. A tourniquet using a soft cotton band is applied, the skin is disinfected, and a 21–23 Gauge needle is used for the blood sampling. The infant will be monitored until the treatment is concluded. In case of failed attempts, only the first venipuncture will be used in the study. In the breastfeeding condition, the midwife/nurse will make sure that the infant is latched and sucking well before the blood test is performed to safeguard the analgesic effect of breastfeeding. The infants’ behavioral responses to pain together with oxygen saturation and heart rate values are videotaped with two digital video cameras and will be assessed subsequently from the video films.

### Data analyses, management and monitoring

Methods for statistical analyses are chosen depending on scales of measurement and normal distribution. The data will be analyzed with descriptive and comparative statistics. Continuous variables that are normally distribute will be presented with mean and standard deviation and compared with t-test for two groups and ANOVA for multiple groups. For data that doesn’t fulfil those criteria we will use median with interquartile range and Mann-Whitney U-test or Kruskall-Wallis one way analysis of variance, respectively. Intention-to-treat will be used, including all randomized parent-infant dyads in the analysis. The division of group 2 into 2a and 2b, which is a nomenclature to distinguish breastfeeding from no breastfeeding, will be taken into account in the comparative analyses and differences between the two subgroups will be scrutinized. Cohens kappa will be used to calculate interrater reliability between PIPP-R scorers.

The data collected in the study is safely stored on a secure server at Örebro University. The project folder on the server is only available to the researchers in the project and is automatically backed-up. The principal investigator is responsible for the data monitoring, ensuring that the researchers in the study are carrying out the research according to the research protocol, ensuring intervention fidelity in the live parental lullaby singing via recurring spot checks with microanalysis of the live singing, ensuring the research is keeping to time and budget and that the research is being conducted ethically. The research group will meet regularly to ensure consistency. During the original development of the project proposal of the SWEpap-study, the research group consulted with parents with previous extensive experience of NICU care. These parents will continue to give feedback on demand.

### Adverse events

Adverse events are not collected specifically but noted when observed and reported to the research team for discussion. Since parent-driven interventions are non-pharmacological, safe and have no known side effects we are not expecting any adverse events.

### Ethics and dissemination

The study is approved by the Swedish Ethical Review Authority (Dnr 2020–01562) and registered at ClinicalTrials. Gov (NCT04341194) in April 2020. Parents will receive both written and verbal information about the study. Written informed consent will be acquired from both parents of each participant. The Swedish Patient Injury Insurance is valid. The parent-driven interventions are non-pharmacological, safe and have no known side effects. The study complies with the ethical conduct of neonatal pain trials [57] by providing pain management for all involved infants and avoiding separation of the newborn infant and her/his parent. The results from the SWEpap-study will be published in scientific journals, in a doctoral thesis and presented at scientific conferences. In order to make our research more accessible to the public, the results will also be presented at health care related workshops and seminars, published in popular science journals and family and parenting magazines and communicated with the media (newspapers, radio, television and social media).

## Discussion

This study protocol describes a randomized controlled trial where the pain reliving effects of combined parent-driven pain management during venipuncture on newborn infants will be studied. Parents are a valuable resource in procedural pain management in newborn infants, a resource which has not been addressed in research until the last decade. In the SWEpap study, we emphasize the importance of parent involvement in newborn pain management, but the key question is to address the research gap in the literature of the efficacy of combined non-pharmacological parent-driven pain management. Combined parental soothing behaviors that provide rhythmic (holding/rocking/vocalizing) or orogustatory/orotactile (feeding/pacifying) stimulation that keep the parent close to the infant, are effective in a painful context [58]. Consequently, in the SWEpap study, we include parental live lullaby singing, which is an unexplored but promising biopsychosocial, multimodal and multisensory pain alleviating adjuvant, especially in combination with SSC and breastfeeding [37]. Newborn infants are highly responsive to social and affective communication. In live parental infant-directed singing, the parent is attuned to the moment-to-moment psychological experience of the infant [43, 59]. The mainly “physical” parent-driven interventions with SSC and breastfeeding are therefore accompanied by a relationship-based intervention, the live parental lullaby singing, which may assist in modifying the painful situation for both the infant and the parent before, during and after the painful procedure [37].

In research, SSC is most often provided by mothers [60]. There are few studies exploring gender differences, mothers versus fathers, or pain alleviating differences in SSC between the mother and her partner or a non-related woman. When testing paternal versus maternal SSC to reduce pain from heel lance, mothers were marginally more effective than fathers in decreasing pain response [61]. Non-related women who provide SSC for procedural pain in preterm neonates are marginally less effective than mothers at decreasing pain response [60, 62].

Within the interdisciplinary research field of neonatal pain, there is a small number of research studies that have addressed the parents’ voice and musical presence during painful procedures [63, 64]. However, the nature of the parents’ vocal and musical engagement with their infants was not systematically reported. The parental live singing was not described and differentiated from “babytalk”, reciting of nursery rhymes or reading a story [64]. In general, live infant-directed singing is considerably more effective than infant-directed speech in lowering infants’ elevated arousal levels and ameliorating distress [65]. Analyses of parents’ singing to their infant in a non-painful context, have revealed similarities in the singing style of mothers and fathers, for example fathers, like mothers, adjust their singing with slower tempo and with warmer vocal tone when singing to infants than in other contexts [66, 67]. The soothing, comforting and emotion regulating properties of a lullaby are well-known cross-culturally and historically [65]. To date, research about parental infant-directed singing has focused primarily on the importance of the mother’s voice, but the possibility for fathers and partners to partake in live lullaby singing with their infant may facilitate a more equal parenthood [68, 69].

In the SWEpap study, multiple methods will be used when assessing the infants’ pain; GSR which comprises neurophysiological signs of pain and stress, and PIPP-R which is a widely used and validated pain assessment instrument. In addition, according to the parent-driven practice where parents are integrated in procedural support, the parents in the SWEpap study will also be assessing their infant’s pain. Pain assessment is as subjective as the pain experience itself and it is important to recognize the reciprocal relationship between infant and parent in a pain assessment context [39]. Parents are acknowledged as having a crucial role in assessing the infant’s pain and taking appropriate action to manage it [70]. Research has also shown that the parent’s assessment is not always based on the infant’s behavior [71] and may therefore differ from the health care professionals’ assessment [72].

To reduce the risk for selection bias in this study, broad inclusion criteria will be used including also parent-infant dyads from other cultures who understands English or Swedish, which strengthens the results and generalizability. A strength in this study is that data collection will take place in four hospitals in diverse health care regions in Sweden which will hopefully increase the scientific quality by recruiting a large sample size within a feasible time period, increasing the generalizability to other settings and reduce the risk of recruitment bias. The allocation with group 2a and b, referring to two different groups receiving skin-to-skin contact, might be considered as problematic. However, performing clinical research sometimes makes it difficult to use standardized procedures. It is not ethically feasible to defer from already existing guidelines and, in this study, preventing mothers from breastfeeding if applicable. It is important to focus on relevant clinical situations where the results can be useful for the patients, families and various health care settings and not only conducting research in laboratory surroundings. An aspect that must be taken into consideration is how to empower parents to use their voice and sing in conjunction with painful procedures. Parents might feel shy and inhibited about singing, especially in unfamiliar situations among unfamiliar people. On the other hand, research shows that most parents (60%) already sing spontaneously to their newborn during hospitalization [73]. By starting the lullaby singing together with SSC 10 min before the venipuncture and giving the parents clear instructions how the singing should be performed, we might modulate possible concerns. The use of technical equipment during the venipuncture will add a few minutes to the procedure, however the research group has previous extensive experience of similar research projects that will ensure the implementation of the study protocol.

The results generated in the SWEpap study will hopefully contribute to the interdisciplinary endeavor world-wide of involving and integrating parents in neonatal pain management and presumably also inform pain management practice in the neonatal intensive care context, where the critically ill and vulnerable hospitalized infants suffer the most from repeated, cumulative and inadequately treated procedural pain in addition to separation from the parents.

## Data Availability

The datasets used and/or analyzed during the current study are available from the corresponding author on reasonable request.

## Abbreviations

FCC: Family-centered care
GSR: Galvanic skin response
NICU: Neonatal intensive care unit
PIPP-R: Premature infant pain profile-revised
RCT: Randomized controlled trial
SSC: Skin to skin contact
PIDS: Live parental infant-directed lullaby singing
VAS: Visual analogue scale
